# Toward Precision Psychiatry in Bipolar Disorder: Staging 2.0

**DOI:** 10.3389/fpsyt.2018.00641

**Published:** 2018-11-29

**Authors:** Estela Salagre, Seetal Dodd, Alberto Aedo, Adriane Rosa, Silvia Amoretti, Justo Pinzon, Maria Reinares, Michael Berk, Flavio Pereira Kapczinski, Eduard Vieta, Iria Grande

**Affiliations:** ^1^Barcelona Bipolar Disorders Program, Hospital Clinic, Institute of Neurosciences, University of Barcelona, IDIBAPS, CIBERSAM, Barcelona, Spain; ^2^IMPACT Strategic Research Centre, Barwon Health, Deakin University, Geelong, VIC, Australia; ^3^Department of Psychiatry, University of Melbourne, Parkville, VIC, Australia; ^4^Orygen, The National Centre of Excellence in Youth Mental Health, Melbourne, VIC, Australia; ^5^Bipolar Disorders Unit, Department of Psychiatry, School of Medicine, Pontificia Universidad Católica de Chile, Santiago, Chile; ^6^Laboratory of Molecular Psychiatry, Hospital de Clínicas de Porto Alegre, Porto Alegre, Brazil; ^7^Postgraduate Program: Psychiatry and Behavioral Science, Universidade Federal do Rio Grande do Sul (UFRGS), Porto Alegre, Brazil; ^8^Department of Pharmacology and Postgraduate Program: Pharmacology and Therapeutics, Universidade Federal do Rio Grande do Sul (UFRGS), Porto Alegre, Brazil; ^9^Barcelona Clínic Schizophrenia Unit, Hospital Clinic de Barcelona, CIBERSAM, Barcelona, Spain; ^10^Florey Institute for Neuroscience and Mental Health, Parkville, VIC, Australia; ^11^Department of Psychiatry & Behavioral Neurosciences, Mcmaster University, Hamilton, ON, Canada

**Keywords:** bipolar disorder, staging, biomarkers, personalized psychiatry, prevention

## Abstract

Personalized treatment is defined as choosing the “right treatment for the right person at the right time.” Although psychiatry has not yet reached this level of precision, we are on the way thanks to recent technological developments that may aid to detect plausible molecular and genetic markers. At the moment there are some models that are contributing to precision psychiatry through the concept of staging. While staging was initially presented as a way to categorize patients according to clinical presentation, course, and illness severity, current staging models integrate multiple levels of information that can help to define each patient's characteristics, severity, and prognosis in a more precise and individualized way. Moreover, staging might serve as the foundation to create a clinical decision-making algorithm on the basis of the patient's stage. In this review we will summarize the evolution of the bipolar disorder staging model in relation to the new discoveries on the neurobiology of bipolar disorder. Furthermore, we will discuss how the latest and future progress in psychiatry might transform current staging models into precision staging models.

## Introduction

Bipolar disorder is a chronic psychiatric condition characterized by mood swings with both manic and depressive symptoms (1). Despite this general picture, bipolar disorder is a highly heterogeneous condition regarding clinical presentation, response to treatment and functional outcome (2, 3). Subsequent DSM and ICD versions have increasingly reflected this heterogeneity, for instance, by adding diagnosis and course specifiers (4). Still, the focus of current systems of classification remains largely cross-sectional and limited to clinical features (5). Moreover, these criteria apply to people with established disorder, but miss people in the prodromal phases of the illness (4).

Emerging data points to the need of a broader approach to bipolar disorder. There is increasing evidence that bipolar disorder is a neuroprogressive disorder, meaning that longer duration of the disease entails more pronounced changes at the clinical and neuropathological level, which may lead to treatment refractoriness and neuropsychological deficits (6, 7). Moreover, several studies support the notion of a prodromal stage before illness onset (8). In an attempt to introduce a longitudinal perspective of the illness in the diagnostic process which would include the earliest phases of bipolar disorder and guide treatment and prognosis, some authors have suggested incorporating the staging model in psychiatry (9–13).

The staging model is based on the concept that an illness progresses following an identifiable temporal progression, from at-risk or prodromal stages to chronic ones (10). Moreover, considering the neuroprogressive course of psychiatric disorders, the staging model assumes that treatment needs and response may differ according to stage. While early stages of the disease might show a better response to simpler treatment regimens, chronic stages might need more complex treatments and still show less clinical improvement (14). Consequently, defining the stage in which the patient is located may help clinicians to choose the treatment that is better adapted to the patient's needs (14). Additionally, the administration of a timely treatment precisely adapted to the stage in which the patient is located might modify or even prevent the progression to subsequent stages of the disease (10).

The staging model in bipolar disorder has been in constant development since its introduction in psychiatry. As new evidence on bipolar disorder has emerged, staging models were refined according to these new findings. In spite of this, experts supporting the staging model still warn that this model gives a standard vision of the progression of the disorder that might not suit every patient (15, 16).

New advances in the field of biological markers (e.g., molecular and neuroanatomical markers of illness vulnerability and/or progression), genetics (e.g., genetic markers or pharmacogenomics) or computer science (e.g., machine learning approaches) might provide current staging models of a higher level of precision regarding diagnosis, prognosis, and treatment choice (15, 16), allowing a more personalized approach to the patient.

The aim of this review is to summarize the evolution of the staging model in bipolar disorder in relation to the new discoveries on the course and neurobiology of the disease. Furthermore, we will discuss how the latest and ongoing progresses in psychiatry might transform current staging models into precision staging models.

## The evolution of staging models in bipolar disorder

### The dawn of staging in psychiatry: fava and kellner staging model (1993)

Fava and Kellner, in 1993, first proposed the application of the concept of staging to psychiatric disorders (9), as staging had shown to be useful in other complex diseases potentially severe if untreated, such as diabetes mellitus, cardiovascular diseases and neoplastic diseases. However, their staging model faced a major limitation in psychiatry research, which was the dearth of longitudinal studies assessing the progression of psychiatric disorders and the scarce data available on prodromal symptoms.

As a result, the staging model proposed by Fava and Kellner did not focus on the longitudinal course of bipolar disorder, but described the different stages that can be seen in a manic episode based on symptom severity (Table 1). Although their model referred only to the manic phase of the disease, Fava and Kellner provided the basis for future staging models in psychiatry.

**Table 1:** Stage definition according to each staging model.

	**Clinical stage definition**				
	**Fava and Kellner (****9****)**	**McGorry et al. (****10****)**	**Berk et al. (****11****)**	**Kapczinski et al. (****12****)**	**Duffy (****13****)**
At-risk stages	**Stage 1:** Prodromal manic symptoms (increased self-confidence, energy and elated mood)	**Stage 0:** Increased risk of psychotic or severe mood disorder without symptoms	**Stage 0:** Increased risk of severe mood disorder (e.g., family history, abuse, substance use)	**Latent Stage:** At risk for developing BD, positive family history, mood or anxiety symptoms without criteria for threshold BD	**Stage 0: a) Classical Episodic Bipolar:** asymptomatic individuals + familial risk for classical BD or recurrent affective disorders **b) Spectrum bipolar:** asymptomatic individuals + familial risk for chronic psychotic disorders or atypical bipolar disorder.
		*BIOMARKERS: Trait marker candidates and endophenotypes*, e.g., *Smooth Pursuit Eye Movements, P 50, Niacin sensitivity, Binocular rivalry, Prepulse Inhibition, Mismatch Negativity, Olfactory deficits, etc*.	No specific symptoms currently	
		**Stage 1a:** Mild or non-specific symptoms, mild functional change or decline	**Stage 1a:** Mild or non-specific symptoms of mood disorder		**Stage 1: a) Classical Episodic Bipolar:** non-specific syndromes + familial risk for classical BD or recurrent affective disorders. **b) Spectrum bipolar:** non-specific syndromes and neurodevelopmental disorders + familial risk for chronic psychotic disorders or atypical BD.
		**Stage 1b:** Ultra high risk: moderate but subthreshold symptoms, moderate neurocognitive changes and functional decline (GAF < 70) *BIOMARKERS: Niacin sensitivity, folate status, MRI and MRS changes, HPA axis dysregulation*	**Stage 1b:** Prodromal features: ultra-high risk	
Early stages	**Stage 2:** Hypomania	**Stage 2:** First episode of psychotic or severe mood disorder Full threshold disorder with moderate-severe symptoms, neurocognitive deficits and functional decline (GAF 30-50)	**Stage 2:** First-episode threshold mood disorder	**Stage I**: Well-defined periods of euthymia without overt psychiatric symptoms *BIOMARKERS: ↑ TNF-α, ↓ 3-NT*	**Stage 2: a) Classical Episodic Bipolar:** minor mood and single episode depressive disorder + familial risk for classical BD or recurrent affective disorders. **b) Spectrum bipolar disorder:** negative syndrome + familial risk for chronic psychotic disorders or atypical BD.
Mid-stages	**Stage 3:** Manic episode without psychotic features	**Stage 3a:** Incomplete remission from first episode	**Stage 3a:** Recurrence of sub-threshold mood symptoms	**Stage II:** Symptoms in interepisodic periods related to comorbidities *BIOMARKERS: ↓ TNF-α, ↓ BDNF, ↑ 3-NT*	**Stage 3: a) Classical Episodic Bipolar:** recurrent major depressive disorder. **b) Spectrum Bipolar:** attenuated psychotic syndrome.
		**Stage 3b:** Recurrence/relapse of psychotic/mood disorder which stabilizes with treatment at a level of GAF, residual symptoms, or neurocognition below the best level achieved following remission from first episode	**Stage 3b:** First threshold relapse	
		**Stage 3c:** Specialist care services	**Stage 3c:** Multiple relapses	
Late stages	**Stage 4:** Manic episode with psychotic features	**Stage 4:** Severe, persistent or unremitting illness as judged on symptoms, neurocognition and disability criteria.	**Stage 4:** Persistent unremitting illness	**Stage III:** Marked impairment in cognition and functioning *BIOMARKERS: Morphometric changes in brain may be present, ↑ TNF-α, ↓BDNF, ↑ 3-NT*	**Stage 4a: a) Classical Episodic Bipolar:** classic BD. **b) Spectrum bipolar:** mixed mania, psychotic/cyclic mania
					**Stage 4b: a) Classical Episodic Bipolar:** BD with residual symptoms. **b) Spectrum bipolar:** psychotic disorders.

*BD, bipolar disorder; BDNF, brain-derived neurotrophic factor; GAF, Global Assessment of Functioning; HPA, hypothalamic–pituitary–adrenal; MRI, magnetic resonance imaging; MRS, magnetic resonance spectroscopy; TNF-α, tumor necrosis factor alpha; 3-NT, 3-nytrotyrosine*.

### The spread of the concept of staging in psychiatry: McGorry et al. (10)

In 2006, McGorry and colleagues introduced a staging model which highlighted the longitudinal course of psychiatric diseases in the psychotic spectrum, also integrating mood disorders (10). They underlined that the staging model does not imply that every patient needs to go through every stage. The main characteristic of McGorry and colleagues' model is that it is built on evidence on major psychiatric disorders jointly and not exclusively on data on bipolar disorder. Importantly, compiling evidence emerging from research on neurobiological correlates of psychotic disorders, allowed McGorry and colleagues to go one step forward and include some biological and endophenotypic markers in the earlier stages of their model (Table 1). They warned, though, that evidence on biological markers arose from studies that evaluated patients with long-established disease, raising the question whether these biological markers were inherent to psychiatric disorders or a consequence of illness duration.

They also incorporated some indicators of illness extent and progression -that is, functioning and cognitive impairment- in their staging model. They defended the importance of addressing social adaptation when assessing patients, as they noted that a person who already presents a great deal of collateral academic or social damage at illness onset may be less likely to respond to treatment and hence is more prone to have a worse prognosis. McGorry and colleagues have continued to progress a transdiagnostic staging model, arguing that the early stages are non-specific, although the later courses of different major psychiatric disorders can have divergent course and outcome patterns (17).

### New insights on bipolar disorder progression: berk et al. (11)

Although similar to and adapting from McGorry and colleagues' model (10), Berk and colleagues' model focused exclusively on bipolar disorder (11). At that moment, a growing body of evidence on a prodromal state for bipolar disorder started to appear (11). Besides identifying risk factors for bipolar disorder, mainly a positive family history of mood disorder and stressful life events (18–20), emerging studies on high-risk youth described a series of prodromal symptoms (21–23), therefore supporting the notion of a traceable at-risk stage.

Moreover, at that moment there was increasing evidence emerging from clinical, neuroimaging and neurocognitive studies that supported a progressive and deteriorating course of bipolar disorder (11). For instance, it had been reported that inter-episode periods were longer after the first episodes, but tended to shorten as the number of episodes increased (24). It had also been found that longer duration of the illness with multiple relapses seemed to be associated with increased medical comorbidities and increased suicidal risk (25). Furthermore, available evidence suggested that response to psychological and pharmacological treatments might not be the same over the illness course (26–29). Response to lithium, for example, seemed to be better if started early after illness onset (30, 31) and before multiple relapses had taken place (32). The number of episodes had also been found to be related to neuroanatomic changes in the brain (33). In 2002, Strakowski et al. (33) described increased lateral ventricular size in bipolar patients with multiple manic episodes, but not in first-episode patients. Likewise, evidence supported that a longer duration of illness and a larger number of episodes was associated with cognitive dysfunction which, in turn, seemed to involve a worse clinical course and functional disability (34). The authors hypothesized that all those alterations observed in the later stages of bipolar disorder were a consequence of progressive changes in the central nervous system due to subsequent mood episodes (6, 7). This phenomenon was called neuroprogression (6). Berk and colleagues suggested several possible pathways involved in neuroprogression including inflammation, oxidative stress, neurotrophins imbalance, mitochondrial dysfunction and epigenetics (6, 7).

Drawing all this evidence together, Berk and colleagues described a staging model with a special focus on the initial phases of the disease and number of episodes (Table 1).

### The ascendance of biological psychiatry: Kapczinski et al. (12)

Kapczinski and colleagues' model appeared at a moment when biological explanations gained prominence and risk phases were explained based on a gene-environmental (GxE) approach (12). For early stages, the GxE perspective suggested that individual genetic differences determine distinct resilience or vulnerability to environmental stress, placing individuals at different risk levels to develop bipolar disorder (35, 36). For late stages, this approach suggested that every individual has a different neuronal resilience to the deleterious effect of repetitive mood episodes (12). Along this line, Kapczinski et al. (37) adapted McEwens' notion of allostatic load to bipolar disorder (38). This concept implies that the interaction of neuroprogressive changes, somatic comorbidities and substance abuse leads to a dwindling resilience to life stress, especially if coping skills are poor (37). Hence, according to Post's kindling hypothesis (36), while stressful life events are an important trigger for first affective episodes, later on the course of the disease recurrences might take place without a clear environmental factor (37).

At that time, studies focusing on the pathophysiology of bipolar disorder reported a deregulation of oxidative and inflammatory pathways in bipolar disorder, especially during mood episodes (39–43), which came with a decrease in neurotrophic factors, like brain-derived neurotrophic factor (BDNF) (44–46). Importantly, it was also described that levels of neurotrophins, oxidative and inflammatory markers differed depending on illness stage (47, 48). For instance, compared to controls, the serum levels of the pro-inflammatory cytokine IL-6 were increased both in the early and late stages of bipolar disorder, while levels of BDNF and the anti-inflammatory cytokine IL-10 were decreased in late stages (meaning patients with 10–20 years of illness duration) but not in early stages (47). TNF-alpha levels appeared to be elevated throughout the illness course but were even higher in later stages (47). In addition, some parameters of oxidative stress, such as 3-nitrotyrosine, were found to be altered in the early and late stages of bipolar disorder, but not in controls (48). The activity of key enzymes in the glutathione pathway was found to be increased in late-stage patients compared with early-stage patients and controls (48). Hence, these data supported the hypothesis presented by Berk and colleagues indicating that neurotrophic, inflammatory and oxidative pathways may be involved in neuroprogression (6). Furthermore, neuroimaging findings also supported the concept of neuroprogression, as although some cerebral structures were shown to be already altered in early stages (49–51), longitudinal studies indicated that patients with repetitive mood episodes showed a progressive brain gray matter loss (52, 53). All these findings implied the identification of putative biomarkers that could be useful to distinguish between patients in early and late stages of bipolar disorder (54).

Psychosocial functioning was also gaining momentum as an outcome measure in bipolar disorder, since it had been demonstrated that symptomatic recovery is not equivalent to functional recovery (55). Psychosocial functioning involves domains such as work and education, leisure time, social and affective relationships or independent living (56), and it can be negatively affected by clinical variables and neurocognitive impairments (57).

Accordingly, Kapczinski and colleagues presented a model based on functioning that, moreover, incorporated cognition and biomarkers (12) (Table 1).

### A broader vision of bipolar disorder: duffy (13)

Duffy proposed a more integrative clinical staging model which described the natural history of bipolar disorder according to illness subtypes: the classical form of bipolar disorder (alternant manic-depressive episodes) vs. the broader bipolar spectrum (13). Duffy claimed that, while the classical form of bipolar disorder tended to follow the progressive course described in previous staging models (i.e., a recurrent and deteriorating course with an increasingly shorter inter-episodic period), other subtypes of bipolar disorder might present a different evolution (13). Evidence, for instance, supported that lithium non-responders showed a more chronic course and a higher-risk of non-affective disorders in family members (58, 59). Neuroimaging and genetic differences between classical lithium responsive bipolar patients and lithium non-responsive bipolar patients were also reported (60, 61). Moreover, her model was supported by longitudinal data showing differences between offspring of lithium responders and lithium non-responders regarding the prodromal period and longitudinal course of bipolar disorder. Offspring of lithium responders had a personal history of anxiety and sleep disorders before illness onset and, once bipolar disorder was established, tended to show an episodic remitting course with good response to lithium (62–64). In contrast, offspring of lithium non-responders manifested higher rates of early developmental alterations, attention deficits and cluster A personality traits (62–64) and, for those who developed bipolar disorder, illness course tended to be more torpid and response to anticonvulsant or atypical antipsychotic seemed to be better than to lithium (63). Thus, Duffy aimed to present an integrative staging model that describes the expected longitudinal course of classical episodic bipolar disorder and of bipolar spectrum disorder (Table 1).

## When staging is not enough

Although different staging models have been proposed in bipolar disorder over the last 25 years, they still need to be better operationalized and validated by empirical research (14). The idea behind the different staging models is to allow defining, for every individual, the extent of illness progression in the moment of the evaluation (65). This can help to refine diagnosis, adjust prognosis and choose the best treatment according to illness stage (66). In this regard, authors have suggested some treatment approaches adapted to every stage: most models agree that prodromal stages would benefit from interventions targeted toward reducing stressors and increasing coping skills; early stages would benefit from patient and family psychoeducation and simpler pharmacological regimens; while mid-stages would need more intensive psychotherapies and more complex pharmacotherapies (12, 15). Clozapine or functional remediation therapies would be reserved for more chronic stages (15). Some individuals with highly refractory illness may need more “palliative” approaches focusing on reduction of side-effects and unnecessary polypharmacy, limited symptom control, identifying and targeting psychological and social problems, and setting realistic goals to aim for the best quality of life for people and their families within the envelope of their disability (67).

**Figure 1:**
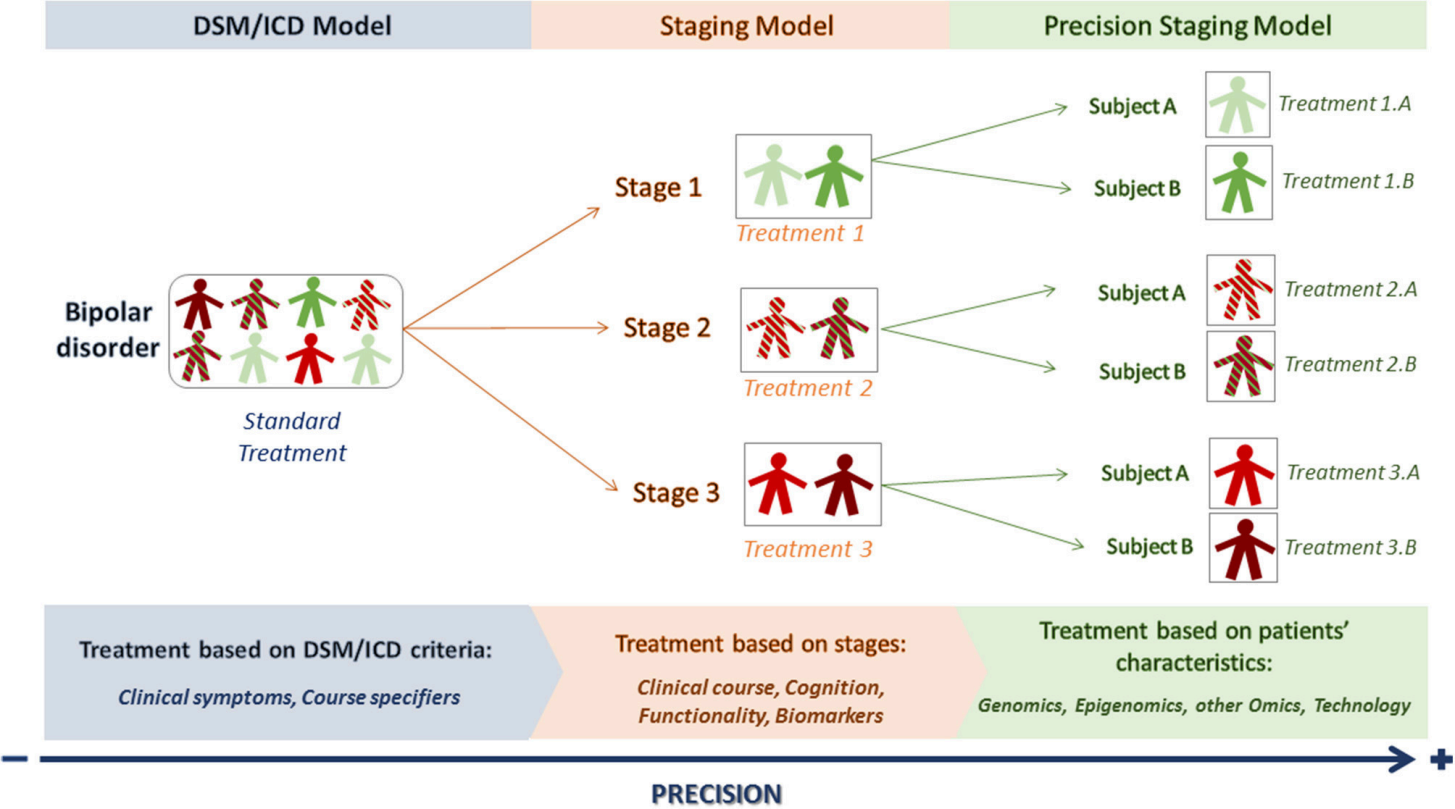
From DSM/ICD to precision staging models. The DSM/ICD model classifies patients into particular conditions according to clinical-based criteria. DSM/ICD diagnosis can be refined by course specifiers. The staging model allows placing the patient in a particular stage according to the extent of illness progression, starting from at-risk stages (stage 1) to more chronic ones (stage 3). However, there can still be differences between patients within a particular stage. A precision staging model would then use the new advances on precision medicine to better characterize the patients and offer them a more personalized treatment.

However, even if the staging model proposes stage-targeted treatments that might provide a better clinical outcome with less side effects, there are still differences among the patients of a particular stage. In consequence, “standard stage-adapted treatments” may not be useful for every patient at a particular stage (15) and increasing the level of precision in every stage would be desirable in order to achieve an even more personalized way of approaching the patient (16) (Figure 1).

## From staging to precision staging models

The aim of precision psychiatry is to offer the patient tailored medical decisions and treatments (68). For that purpose, precision psychiatry needs to integrate biographical, clinical and biological information regarding each individual (69). In addition, precision psychiatry is envisaged to benefit from the coming advances in technological, data, and computer science to aid diagnostic processes and treatment provision. A precision staging model would ideally incorporate all these recent progresses into the appropriate stage (Table 2).

**Table 2:** Potential precision staging model in bipolar disorder.

**Clinical stage**	**Definition**	**Potential precision tools and precision interventions**	**Domains to be assessed and general interventions**
At-risk stages	Increased risk of severe mood disorder (e.g., family history, abuse, substance use) Asymptomatic or non-specific symptoms of mood disorder	Individualized evaluation of risk/protective factors Genetic and epigenetic markers	a) Clinical domain b) Cognitive domain c) Functional domain d) Comorbidities domain: - Substance use - Physical comorbidities - Psychological comorbidities
	Prodromal features: ultra-high risk	Genetic and epigenetic markers Risk Calculator Risk biomarkers (molecular, neuroimaging) Machine learning approaches (risk for bipolar disorder) Cognitive enhancers	
Early stages	First-episode threshold mood disorder	Genetic markers of treatment response Epigenetics (illness course) Biomarkers of treatment response and illness progression Machine learning approaches (suicide risk) mHealth (Psychoeducation, monitoring)	Pharmacotherapy and psychological interventions adapted to each individual physical and psychological comorbidities
Mid stages	Clinical relapse	Pharmacogenetic tests Biomarkers of treatment response and illness progression Machine learning approaches (suicide risk) mHealth (psychoeducation, monitoring) Functional remediation tailored to patients' profile, cognitive enhancers	Substance use intervention
Late stages	Persistent unremitting illness	Functional remediation tailored to patients' profile	

Many advances in precision medicine are related to genomics. Genomics have led to improvements in staging models in some branches of medicine, especially cancer (70). However, psychiatric disorders are genetically complex conditions and their genetic underpinnings remain to be determined. Still, international consortia that comprise samples from several countries have brought some light on risk loci associated with bipolar disorder (71, 72). These collaborative genome-wide association studies (GWAS) allow overcoming replication difficulties often seen in genetic studies due to small sample sizes. One of these studies analyzed genomic data on a sample of 40,000 bipolar patients and replicated the discoveries of previous GWAS studies regarding several single-nucleotide polymorphisms (SNPs) statistically associated with the disease, including variants within the genes CACNA1C, ANK3, MAD1L1, and SYNE1 (73). Two new risk loci were also identified (73). Moreover, the Psychiatric Genomics Consortium has recently identified specific loci that distinguish between bipolar disorder and schizophrenia (74). Genetic markers promise to be valuable at the earliest stages of bipolar disorder, as the main aim of mental health approaches at at-risk and early stages is to predict disease vulnerability and make accurate diagnosis. So far, though, little of the advances in genomics have translated into clinically useful tools.

Besides genetic markers, screening for risk factors and epigenetic modifications may be another useful tool at at-risk stages of the disease, given that stressful life events, particularly childhood trauma, can alter DNA methylation and may increase the risk of developing mood disorders (75). Concordant with this, childhood verbal, physical, or sexual abuse has been related to a worse illness course (76).

Risk calculators are another promising tool for at-risk stages (77), as the multifactorial and polygenic nature of bipolar disorder makes it improbable that a single factor can accurately predict its onset (78). The Pittsburgh Bipolar Offspring Study group (BIOS) has recently developed a risk calculator to predict the 5-year risk of bipolar disorder onset in offspring of parents with bipolar disorder combining dimensional measures of mania, depression, anxiety, mood lability, psychosocial functioning, and parental age of mood disorder onset (79). Although their findings need to be replicated, the model seemed to be able to predict onset of bipolar disorder with an area under the curve (AUC) in the receiver operating characteristic curve analysis of 0.76 and might be of potential value for youth at ultra-high risk for BD. Machine learning, a field of computer science that studies and constructs algorithms that can learn from large number of data, find patterns and make predictions (80), might also be useful to estimate the individual probability of a particular outcome (80, 81). Mourao-Miranda et al. (82), for instance, found that machine learning approaches using functional magnetic resonance imaging (fMRI) data could differentiate between adolescents genetically at-risk for mood disorders and healthy controls with a 75% accuracy (sensitivity = 75%, specificity = 75%). Moreover, those at-risk adolescents who developed an anxiety or depressive disorder at follow-up showed significantly higher predictive probabilities, therefore suggesting that predictive probabilities could be used as a score to predict which at-risk adolescents would develop a mood disorder in the future (82).

Early and middle stages of bipolar disorder may benefit from progress in the field of pharmacogenomics. This is the study of genetic variations that affects individual response to drugs and vulnerability to adverse effects (83). After the first acute episode, selecting the best treatment for the patient, both in terms of efficacy and tolerability is a necessary but complex task. International consortiums in genetics, such as the International Consortium of Lithium Genetics (ConLiGen) (71), have worked to disentangle genetic variants associated with treatment response, mainly response to lithium. In 2016, the ConLiGen consortium uniformly phenotyped 2,563 bipolar patients and reported a genome-wide significant association with a locus of four linked SNPs on chromosome 21 and lithium response (84). Another recent GWAS performed by the ConLiGen consortium displayed that bipolar patients with a low genetic load for schizophrenia showed a better response to lithium (85). Pharmacogenetic screening for hepatic cytochrome P450 genetic polymorphisms can also be helpful in the near future to predict tolerability and side effects of psychiatric treatments (86). While the precise patient profile that would benefit from these tests remains to be elucidated, pharmacogenetic tests are kept for selected patients with unusual patterns of drug response or unexpected adverse reactions (83, 87).

Less progress has been made in the field of biological markers in the last few years and data on molecular and neuroimaging biomarkers is still contradictory and limited by the heterogeneity between studies and the poor specificity of the putative biomarkers (88). Although evidence is not yet compelling, some biological markers have been suggested to be associated with increased risk of conversion to bipolar disorder, and therefore may be useful when assessing subjects at at-risk stages. fMRI studies report that frontal hyperactivation during working memory paradigms may be associated with genetic risk for bipolar disorder (89, 90). In more established stages, neuroimaging might be useful to monitor treatment response (91). Also, a preliminary study using a voxel-based morphometry-pattern classification approach was able to distinguish between patients with unipolar and bipolar depression based on structural gray matter differences (92). Studies on biological markers have also suggested that peripheral concentrations of BDNF could be used to discriminate unipolar depression from bipolar depression (93, 94), but evidence is not clear (95). This would be of the utmost importance in the earliest stages of the disease, considering that bipolar disorder is often misdiagnosed since the index episode is frequently depressive. In consequence, patients are treated with antidepressants and the introduction of a mood stabilizer is delayed until the first manic episode is detected, which may negatively affect illness course and prognosis (8).

Regarding other molecular markers, hypothalamic-pituitary-adrenal (HPA) axis dysfunction is thought to be one of the pathways involved in neuroprogression in bipolar disorder (96), but it has also been suggested to be a useful trait marker in high-risk individuals (97, 98). Alterations in neurotransmitters transporters have been suggested as markers of bipolar disorder (96), but there is no evidence on changes in neurotransmitters according to illness stage. Regarding later stages of bipolar disorder, recent studies have reported higher levels of TNF-alpha and IL-6 in late stages of bipolar disorder (99, 100). Similarly, Soeiro-de Souza and colleagues described that patients with recurrent episodes showed increased oxidative and inflammatory markers, which were related to the number of manic episodes (101). Further, increased inflammation, increased oxidative stress and reduced telomere length have been suggested as possible mechanistic links between psychiatric diseases like bipolar disorder and other systemic diseases, such as endocrine or cardiovascular diseases (102–105). Hence, the identification of a deregulation on those pathways related to both psychiatric and somatic diseases may have therapeutic implications (106). For instance, bipolar patients exhibiting persistently increased low-grade inflammation (107) might benefit from anti-inflammatory treatment strategies and from periodic screening of systemic conditions like metabolic syndrome (106). Considering these data, screening for physical comorbidities seems especially important in middle and late stages of the disease, albeit protecting against complications like physical comorbidities or substance abuse should be a priority at every stage of the disease.

Cognition is another important domain that needs an individualized evaluation throughout all the stages of bipolar disorder (108). On one hand, cognitive reserve, defined as the ability of a brain to cope with brain pathology in order to minimize symptoms (109), may be useful in early stages to predict neurocognitive performance in patients with bipolar disorder (110), as it has been found that lower estimated cognitive reserve is associated with worse performance in neuropsychological tests and more functional impairment (110, 111). Similarly, a recent study on first-episode psychosis has found that those patients with affective psychosis with a greater cognitive reserve showed a higher socioeconomic status, better functioning and greater verbal memory performance (112). This study also emphasizes the need to explore the impact of specific interventions, like physical activities and hobbies, on cognitive reserve, since it could be useful to guide the development of personalized treatment programs (112). Therefore, cognitive enhancing strategies might be key in the early stages and not necessarily in the late stages of the disease. On the other hand, evidence points to a heterogeneous cognitive profile in bipolar patients both in “cold” and “hot” cognition (113–115). The presence of such heterogeneous cognitive profiles among patients with bipolar disorder might be taken into account to design more tailored cognitive remediation therapies adapted to each individual needs (116–118). Cognitive deficits may also limit long-term psychosocial functioning, which means that patients with greater cognitive impairment are more likely to experience poorer outcomes. Previously, we showed that patients in stage I and healthy controls had similar functioning patterns. In addition, a strong linear association was found between functioning and clinical stages, suggesting a progressive functional decline from stage I through to stage IV of bipolar disorder. These findings provide further support to the clinical staging model in bipolar disorder, indicating that bipolar patients lie on a continuum of disorder progression ranging from periods of favorable functioning to others of incomplete functional recovery (118). The link between variables related to the course of the illness, cognitive deficits and functioning suggests that early intervention is crucial to prevent illness progression and to improve cognitive/functional outcome. Some studies have also found different profiles of psychosocial functioning in patients with bipolar disorder, which should also be taken into consideration in the framework of a personalized approach (3, 119).

All these advances should complement regular clinical practice, which already contains elements of staging and precision psychiatry. The assessment of the patient's particular symptoms, such as his/her distinctive early signs of relapse, predominant polarity (i.e., the “tendency” to present more depressive or manic relapses) (120) or individual suicide risk (121) is regularly done in clinical settings and is essential to monitor the patient evolution and guide treatment selection. Technological advances used in everyday life, encompassed in the concept of mobile Health (mHealth), might be a valuable tool to help clinicians to collect individualized data on illness course and monitor illness progression (122). For instance, changes in activity, geolocation or sleep patterns may help to detect early signs of mood relapse (123, 124). Additionally, smartphone apps can be used to empower patients with bipolar disorder to detect prodromal symptoms of relapse by providing them personalized psychoeducational messages (125, 126). New methods like machine learning approaches might also be useful in the future to help predict suicide risk (127, 128).

## Discussion

In this review we describe the evolution of the staging model in bipolar disorder since its introduction into psychiatry. The first staging models in bipolar disorder were initially based on evidence derived from cross-sectional studies, but longitudinal studies and data on neuroimaging, peripheral biomarkers, cognition, psychosocial functioning, and prodromal symptoms have successively enriched the staging models (66). We have also described several elements of precision psychiatry that could be incorporated in future precision staging models.

The main advantage of staging and precision medicine is the recognition that a reductionist clinical approach based on the presence or absence of a series of symptoms is not enough to design an adequate therapeutic strategy. These symptoms need to be considered in the light of the illness progression and, most importantly, of the patient's own clinical evolution. For instance, the presence of a switching or non-switching pattern should be considered when evaluating a patient, as it has prognostic implications and therefore might impact staging. As highlighted in a review by Salvadore et al. (129), patients showing a switching pattern [i.e., patients showing a “sudden transition from a mood episode to another episode of the opposite polarity” (129)] usually spend less time in remission, show higher comorbidity rates and substance abuse and are at a higher risk of suicide attempt (129). While mood symptoms will of course still be the cornerstone of bipolar disorder diagnosis, other elements should be likewise considered as they can be as informative as clinical symptoms (9, 10, 12, 15). As such, everyday difficulties, cognitive complains, substance abuse or comorbidities can be markers of illness severity or stage specifiers and merit an individualized assessment and treatment. Social and personal losses due to the illness and previous personality should also be included in a standard evaluation throughout the stages and be given the attention they deserve (10). Patients' insight and perception of the disease should be carefully assessed, as these are important prognosis and therapeutic factors, especially in early stages (8). Medication load, treatment satisfaction, and compliance should be also carefully assessed, as it might influence disease progression. While this way of approaching the patient is naturally adopted by most clinicians and many guidelines, it remains underrepresented in diagnostic manuals (5). In any case, this approach is more in line with the World Health Organization definition of health: “Health is a state of complete physical, mental and social well-being and not merely the absence of disease or infirmity”^1^. The inclusion of self-report measures of well-being in research and clinical care in bipolar disorder may contribute to take into consideration the patient's perspective when assessing the efficacy and usefulness of pharmacological and psychological interventions (130).

Another important point of clinical staging is the assumption that prodromal phases of the disease can be also identified and targeted. The possibility of making an early diagnosis radically changes the way how bipolar disorder in particular, and psychiatric diseases in general, have hitherto been managed. At-risk stages are rather non-specific, though. In consequence, the prodromal period has also been preferably defined as “at risk mental states” (17), as a prodrome is defined as “any symptom that signals the impending onset of a disease” (131) and evidence does not support this definition. On one hand, data from the field of ultra-high risk in psychosis shows that disease onset is not deterministic and a significant proportion of the at-risk youth show a remission of these early symptoms (132). On the other hand, these early symptoms are not specific to any disease but can progress into several possible psychiatric conditions (64). In the absence of specific genetic markers for bipolar disorder or very precise risk calculators, transdiagnostic preventive interventions aimed to reduce stress, educate on mental-well-being and prevent substance abuse are preferable at these at-risk stages (133). Implementing early interventions that include enhancing cognitive reserve by increasing mental stimulation (reading and cognitive exercises), introducing physical exercise and leisure activities or building social skills and social interaction, may provide a set of skills that can help to cope better with the disease (134–136). This kind of preventive interventions or “positive habits” could even be implemented at school or primary care, which could help to reduce stigma on mental health by educating the population on the importance of taking care of mental well-being (133).

In this regard, it has been suggested that a transdiagnostic staging model might be more adequate for the study of at-risk phases, while disorder-specific models are more useful once the fully-develop disorder emerges (15). It is necessary to bear in mind that psychiatric disorders are dynamic and clinical symptoms may evolve over time, requiring a change in diagnosis (137). Nevertheless, the general staging approach supported by stage specifiers should still be useful to assess illness severity regardless of changes in DSM or ICD diagnosis.

Biomarkers also face the problem of lack of specificity. Alterations in the inflammatory or oxidative systems have been found across several psychiatric and medical diagnoses (138). Again, biomarkers could be more stage-specific than illness-specific and be conceived as an additional tool for the assessment of illness risk or treatment outcome. Low sensitivity and replicability seems a bigger handicap. Moreover, most published data on biomarkers are based on the currently commercially available ELISA kits, which is also a limiting factor. State-of-the-art techniques widely used in precision medicine might help to overcome these limitations. A multi-omic approach, meaning using genomic, epigenomics, transcriptomics, proteomics, metabolomics, metagenomics, and lipidomics data, combined with environmental information gathered, for instance, through mobile devices, could help to identify more sensitive biomarkers panels to guide diagnosis and treatment choice (69). However, as these “omic” platforms cannot be used in regular clinical practice, the potential discoveries arising from these platforms need to be translated into an immuno-based assay, which is a more viable option. New strategies with a more integrative approach between clinical factors and biological markers are being proposed in biomarker research of lithium response, which are expected to shed some light on precision drug prescription (139).

Precision in psychiatry implies embracing the multifactoriality of psychiatric diseases and the need to incorporate in the patient's assessment a range of biological and environmental factors that interact with each other in a dynamic way. Moreover, the biological and environmental factors involved in illness onset and progression are particular to every patient, as it is the way they interact (17). The use of personal devices to monitor the trajectories of patients at anytime and anywhere might help to deepen our knowledge on the complex interaction between biological and environmental factors. They can also allow evaluating less studied markers, such as sleep or chronobiological markers, which may turn out to be very informative (140). Moreover, further studies on epigenetics or mitochondrial genomes might identify novel factors involved in this complex disease (141). Similar to what is being developed in the field of psychosis, research on bipolar disorder could benefit from consortia sharing data to develop machine learning algorithms to help the prediction of bipolar disorder onset (17, 142).

A major limitation of current staging models is the absence of an agreement on the definition of stages. Moreover, operationalized cut-off points are lacking, probably due to the lack of longitudinal studies assessing patients according to stages, the absence of clear and reproducible neurobiological markers defining every stage and the intrinsic heterogeneity of psychiatric illnesses (15, 16, 143). Therefore, the current proposed models of staging are mainly theoretical and need to be validated for the moment. Additionally, participants of the available studies assessing differences between early and late stages of bipolar disorder include subjects attending specialized clinics, hence probably representing more severe forms of bipolar disorder (16). Moreover, it should be noted that precision medicine is still in its early beginnings, meaning that findings on genomics, genetic markers, and epigenetics are preliminary and need to be replicated before being integrated in any model of classification.

Until more solid information is available on the biology of the disease, though, the staging models can be based on pragmatic variables, like number of episodes and impact on cognition and functionality. A staging system based on characteristics that can be easily measured allows to standardize it and make it available and applicable in a broader number of clinical settings and countries worldwide (144).

Regardless of what the future brings, personalized medicine means “patient-centered care,” therefore the choice among those new diagnostic techniques or treatments should be subject to a consensus between the clinician and the patient, especially considering the new ethical challenges that precision psychiatry brings with it (145). While psychiatrists can offer their expertise, patients opinions and preferences should play a central role in treatment decisions through shared decision-making (145).

## Author contributions

ES was responsible for conception and design as well as initial drafting of the manuscript. All other authors (SD, AA, AR, SA, JP, MR, MB, FK, EV, and IG) were responsible for revising the manuscript critically for important intellectual content of the version of the manuscript to be published. All authors read and approved the final manuscript.

### Conflict of interest statement

SD has received grants and/or research support from Stanley Medical Research Foundation, Foundation FondaMental, Eli Lilly, GlaxoSmithKline, Organon, Mayne Pharma, and Servier. He has received speaker's fees from Eli Lilly, advisory board fees from Eli Lilly and Novartis and conference travel support from Servier. MB has received grant/research support from the NIH, Cooperative Research Center, Simons Autism Foundation, Cancer Council of Victoria, Stanley Medical Research Foundation, MBF, NHMRC, Beyond Blue, Rotary Health, Geelong Medical Research Foundation, Bristol Myers Squibb, Eli Lilly, Glaxo SmithKline, Meat and Livestock Board, Organon, Novartis, Mayne Pharma, Servier, Woolworths, Avant and the Harry Windsor Foundation, has been a speaker for Astra Zeneca, Bristol Myers Squibb, Eli Lilly, Glaxo SmithKline, Janssen Cilag, Lundbeck, Merck, Pfizer, Sanofi Synthelabo, Servier, Solvay and Wyeth, and served as a consultant to Allergan, Astra Zeneca, Bioadvantex, Bionomics, Collaborative Medicinal Development, Eli Lilly, Grunbiotics, Glaxo SmithKline, Janssen Cilag, LivaNova, Lundbeck, Merck, Mylan, Otsuka, Pfizer, and Servier; FK has received support as a speaker from Janssen and Daiichi-Sankyo in the past 2 years; EV has received grants and served as consultant, advisor or CME speaker for the following entities: AB-Biotics, Allergan, Angelini, AstraZeneca, Bristol-Myers Squibb, Dainippon Sumitomo Pharma, Farmindustria, Ferrer, Forest Research Institute, Gedeon Richter, Glaxo-Smith-Kline, Janssen, Lundbeck, Otsuka, Pfizer, Roche, Sanofi-Aventis, Servier, Shire, Sunovion, Takeda, the Brain and Behavior Foundation, the Spanish Ministry of Science and Innovation (CIBERSAM), the Seventh European Framework Programme (ENBREC), and the Stanley Medical Research Institute; IG has received speaker's fees from Ferrer, Janssen Cilag, and Lundbeck, advisory board fees from Ferrer, Lundbeck, Otsuka, and conference travel support from Lundbeck, Otsuka. The remaining authors declare that the research was conducted in the absence of any commercial or financial relationships that could be construed as a potential conflict of interest.

## Footnotes

^1^WHO. http://apps.who.int/gb/bd/PDF/bd47/EN/constitution-en.pdf?ua=1. 1946
